# Environmental sustainability in diabetes: improving the quality of diabetes management through HTA and system-level change?

**DOI:** 10.1017/S0266462325103206

**Published:** 2025-12-03

**Authors:** Melissa Pegg, Benjamin D. Bray, Mei Sum Chan, Elisabeth de Laguiche, Aparajita Tyagi, Eugenio Di Brino

**Affiliations:** 1 Environmental Impact, York Health Economics Consortium (YHEC), University of York, York, UK; 2 Health Analytics, Lane Clark & Peacock LLP, London, UK; 3 ALTEMS Advisory, Università Cattolica del Sacro Cuore, Rome, Italy; 4 Global Payer Evidence, Novo Nordisk A/S, Søborg; 5 Global HEOR, Novo Nordisk A/S, Søborg

**Keywords:** diabetes management, environmental sustainability, health technology assessment, carbon footprint in health care, sustainable healthcare innovation

## Abstract

Diabetes affects over 500 million people worldwide and contributes substantially to the environmental impact of health care, including carbon emissions and plastic waste. As healthcare systems globally aim to reduce their environmental footprint, there is a need to embed environmental sustainability into decision making and foster innovation in health and life sciences.

This commentary outlines the environmental sustainability challenges and opportunities across the diabetes care pathway, highlighting innovations that reduce the demand for healthcare resources and associated environmental impact. We discuss the current and potential role of health technology assessment (HTA) agencies in promoting more sustainable health systems, by incorporating environmental considerations into the value assessment of technologies. Several approaches, such as integrated and parallel evaluation, are emerging to support this aim, whereas HTA agencies increasingly consider parameters of environmental life cycle assessment (eLCA), a comprehensive framework for evaluating the environmental sustainability of technology. Although a framework is evolving, early implementation by HTA bodies, for example, in the United Kingdom, Thailand, Canada, and Italy, highlights growing momentum. Moreover, sustainability policies at government and health system levels are developing globally, signaling opportunities to incorporate environmental sustainability in HTA (ESHTA).

Given the scale of health care’s environmental footprint, large disease areas offer critical opportunities for sustainable action. Diabetes, with its growing global prevalence, presents a particularly suitable domain for piloting the integration of environmental sustainability into HTA.

## Introduction

The climate crisis is a critical threat to planetary and human health worldwide, and if the rate of global greenhouse gas (GHG) emissions continues, global temperatures are predicted to increase by 5.7 °C above preindustrial temperatures within the next 70 years (1). The consequences of climate change are already evident, including disruptions to food production, reduced water availability and quality, loss of biodiversity, rising morbidity and mortality, and increasing pressures on the resilience of healthcare systems (2;3). Although health care seeks to improve population health, its ecological footprint contributes to adverse health outcomes (2;3). Global health care is responsible for almost 5 percent of total GHG emissions and a similar proportion of air pollutants (2). For example, in 2020 alone, the carbon footprint of the United Kingdom (UK) National Health Service (NHS) was estimated to be 24.9 megatons of carbon dioxide equivalent emissions (CO_2_e) (4).

The majority of jurisdictions (107 countries) have made commitments to achieve net-zero economies (5), whereas several health systems have set net-zero policy targets, including NHS England by 2045 (4). In support of this, the World Health Organization advocates for the integration of environmental sustainability into healthcare policies, including a call for “climate resilient and low carbon infrastructures, technologies and supply chains” (3).

Given the scale and complexity of health care’s environmental footprint, a targeted approach is required to identify practical opportunities for environmental impact reduction. Focusing on specific disease areas allows for a more detailed assessment of the technologies, care pathways, and resource flows that contribute most to emissions and waste, for example. By concentrating efforts within a single clinical domain, such as diabetes, it becomes possible to generate actionable insights, test interventions, and develop models that may later be applied across the wider health system.

Diabetes is one of the most common chronic conditions worldwide, affecting 828 million people aged over 18 years (6). The condition markedly increases the likelihood of serious and often life-altering complications, including amputation, vision loss, and renal failure. It is also strongly linked to a heightened risk of cardiovascular disease, dementia, and infectious diseases such as tuberculosis and severe coronavirus disease 2019 (COVID-19), underscoring its profound and wide-ranging impact on population health (6). Diabetes care typically requires healthcare resource use including monitoring, medication, and device use. This lifelong demand in health care generates substantial resource consumption and waste, making diabetes prevention and care a high-impact area for targeted strategies to reduce health care’s environmental footprint. Given its chronic nature and intensive resource requirements, the evaluation of diabetes interventions offers significant opportunities to identify and reduce environmental impacts across the continuum of its care pathway.

Health technology assessment (HTA) is a multidisciplinary evaluation of the medical, social, economic, organizational, environmental, and ethical issues related to the use of a health technology. An HTA is conducted in a systematic and transparent way to inform decision making, standards, guidance, and policy. As a well-established, evidence-based framework for the impacts of health interventions, HTA offers a structured means of embedding environmental criteria into technology appraisal and adoption processes (7). In this commentary and with reference to the diabetes care pathway, we aim to describe how HTA and the inclusion of environmental sustainability considerations within its framework are well positioned to support sustainable development in health care.

## The environmental impact of the diabetes care pathway

Diabetes represents one of the most prevalent chronic diseases globally, with a rising incidence that places increasing pressure on healthcare systems. Although data on the environmental impact of diabetes are currently limited, the disease area has previously been estimated to contribute to 0.72 megatons of CO_2_e per year in the UK (8), with almost half (44 percent) of the carbon footprint being attributable to the management of diabetes complications (9). This is reflected in the fact that around one in six hospital beds is occupied by someone with diabetes – a known carbon hotspot within the hospital setting (10). Moreover, hospital occupancy as a result of diabetes management is expected to rise to one in four by 2030 (11). Comparably, a large Swedish cohort study with 20 years of follow-up found that individuals with diabetes had approximately double the incidence of hospital admissions compared to nondiabetic peers (12). In addition, patients with diabetes require regular checkups, including specialist consultations (e.g., endocrinologists, ophthalmologists for retinal screening, and podiatrists for foot care) and intensive treatments. One such intensive treatment is hemodialysis, as diabetes is a leading cause of kidney failure. Many patients therefore need to travel to a dialysis center several times per week (13), contributing to the environmental impacts associated with patient travel. In the UK, an analysis of the type 2 diabetes care pathway found that patient travel is one of the top three contributors to the carbon footprint of diabetes care, alongside medication use and inpatient admissions (8).

**Figure 1.**
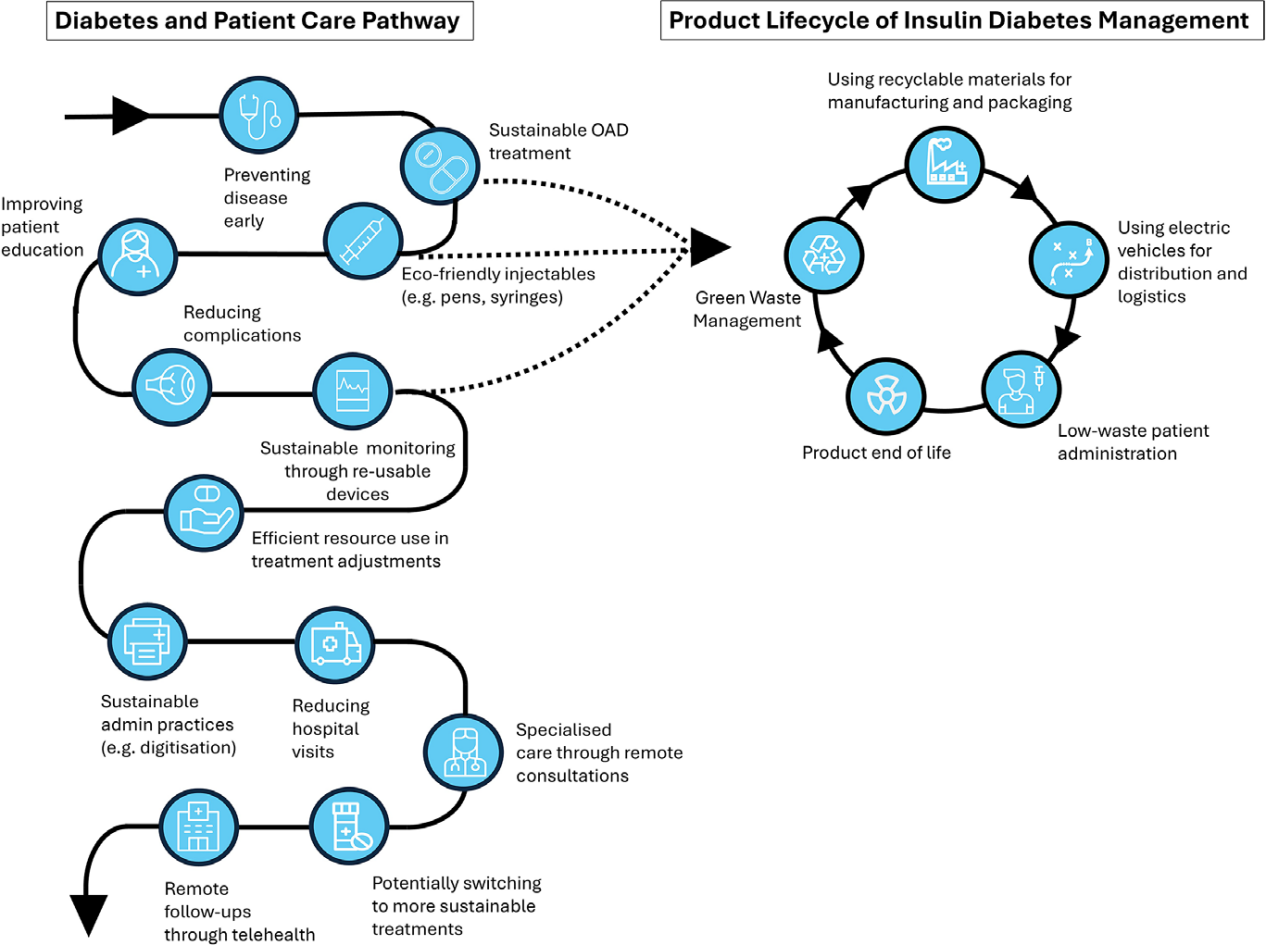
Opportunities for integrating sustainable practices in the diabetes care pathway and insulin product life cycle. Abbreviation: OAD, oral antidiabetic drug.

The environmental burden of diabetes care also stems from the widespread use of disposable medical technologies necessary for day-to-day disease management. Worldwide, an estimated 150–200 million people living with diabetes use insulin and require the use of administration products (14;15). Many of the key components of products used in diabetes management comprise single-use fossil fuel–derived plastics (Figure 1), such as insulin pens, continuous glucose sensors, test strips, and lancets (16;17). For example, the packaging and applicator materials from continuous glucose monitors mainly consist of single-use plastic materials that contribute between 58 and 108 grams of waste per sensor, depending on the device (18). In addition, insulin pumps and pens generate between 1.2 and 1.4 kilograms of plastic and electronic waste per user each month, equivalent to around 2 percent of the total household waste for an average individual in the United States (19). A German clinic also demonstrated that a cohort of 80 insulin users collected a total of 23,707 diabetes-related disposables over a period of three months, estimated to be 1.2 billion discarded items annually (20). Critically, there are broader environmental impacts associated with the diabetes care pathway beyond GHG emissions and the life cycle of plastic materials used in diabetes management. For example, a review of the environmental impact of metformin found measurable concentrations in water treatment systems, seawater, and drinking water, highlighting the growing concern over pharmaceutical pollution in water bodies globally (21).

The burden of diabetes prevalence therefore poses a significant challenge not only to public health but also to planetary health and health care’s ability to reach net-zero targets. Given the substantial environmental impacts across the diabetes care pathway, interventions with greater sustainability potential – such as innovations in health promotion, disease prevention, improved disease management, and optimized resource use – could be evaluated and recommended at different points along the HTA life cycle, while simultaneously enhancing the quality of care.

## Health technology assessment and its potential role in including environmental sustainability

Although health care’s contribution to environmental degradation is increasingly recognized, the integration of environmental sustainability considerations into formal healthcare decision making remains limited. HTA organizations inform healthcare decisions by assessing new and established technologies’ clinical, economic, social, ethical, and sometimes broader domains, such as environmental impacts. They use processes such as defining questions and reviewing evidence, while engaging diverse stakeholder groups throughout the different HTA stages. The HTA process often involves the participation of policy developers, patients, public citizens, healthcare professionals, regulators, health technology developers (HTDs), researchers, and distributors (22). Incorporating multiple viewpoints helps prevent bias in HTA and ensures that both benefits and risks as well as intended and unintended consequences are addressed. Structured consensus methods, including expert panels and other deliberative approaches, are widely used to support policy, reimbursement, and guideline development, providing comprehensive, evidence-based recommendations and standards for regulatory and clinical decision making (22–24).

HTA organizations are well placed to support sustainable healthcare innovation, as they already offer a structured, evidence-based framework that incorporates multiple perspectives. Their outputs are tailored for policy makers, payers, and providers – the same audiences who will need to embed sustainability into procurement, reimbursement, and service delivery decisions. Combined with other policy levers, such as regulation and procurement frameworks, HTA can drive progress toward more sustainable use of health technologies and patient care pathways.

## Maximizing the opportunity to develop sustainable innovation across the diabetes care pathway

Key drivers of sustainable health care across the diabetes care pathway include interventions that (i) prevent the need and reduce the demand for health care in the first place, such as promoting health to prevent the onset of type 2 diabetes and reducing the risk of diabetes complications, and (ii) innovations that reduce the environmental impact of diabetes care itself (Figure 1).

Health promotion and good management of diabetes are cornerstones of sustainable health care, as they reduce the need for resource-intensive treatments, improve population health, and minimize environmental and financial burdens (25). This perspective aligns with the United Nations Sustainable Development Goal target 3.4, to reduce premature mortality from noncommunicable diseases (NCDs) and promote mental health and well-being – a shared societal goal (26). Specifically, prevention programs for type 2 diabetes have been shown to be clinically, economically, and environmentally effective, reducing individual disease risk, delivering cost savings or favorable cost-effectiveness, relieving health system burdens, and reducing environmental impact (9;27). Significant environmental savings can be achieved across health systems by enhancing the quality of care and ensuring that patients receive treatment in accordance with clinical guidelines, while also working to reduce diabetes complications (28). For example, a modeling study estimated that maintaining glycemic control through optimal management of type 2 diabetes (defined as an hemoglobin A1c (HbA1c) level of 7.0 percent) resulted in reduced demand for interventions associated with diabetes complications, saving 1,546 kg CO_2_e per patient (29).

Diabetes care is currently highly resource-intensive, spanning complex treatment pathways that generate a substantial environmental impact. Healthcare systems contribute to this impact through a range of practices, including the use of single-use products within a linear consumption model (“take–make–dispose”). HTDs can play a key role in innovation toward more sustainable care pathways. In support of this, the circular economy represents a widely adopted framework that takes a life cycle perspective, prioritizing the minimization or elimination of produced waste. This can be achieved, for example, by designing out waste, reducing, reusing, and reprocessing products, which can lead to associated reductions in carbon emissions (10). A study applying circular economy in diabetes products compared reusable versus disposable insulin pens and found that reusable pens reduced plastic waste by approximately 89 percent and carbon emissions by around 40 percent (30). Another analysis compared tubeless insulin pumps with different designs, with findings demonstrating that although the modular pump produced slightly more waste by mass equating to 5.5 kg/year vs 4.9 kg, it had a lower global warming impact corresponding to 13.6 vs 15.5 kg CO₂e/year, due to greater recyclability (31). These examples highlight how device design and material choices influence environmental outcomes and support the value of embedding circular economy principles into product development.

Healthcare professionals are increasingly advocating for the adoption of more sustainable practices, recognizing the sector’s significant environmental impact and its implications for patient and population health (10). The Green Surgery report, endorsed by 23 internationally recognized healthcare professional bodies – including NHS England, NHS Scotland, Royal College of Surgeons of England, Royal College of Physicians and Surgeons of Glasgow, and Royal College of Nursing – advocates that HTDs adopt circular economy principles within their operations (10). In support of the principles of circular economy and mitigation of product carbon, several pilot studies illustrate opportunities to utilize more sustainable product innovations in diabetes care, including reusable insulin delivery pens (30) and recycling schemes for insulin delivery pens (17). Aligned with these principles, Denmark’s Returpen™ insulin pen take-back program achieved approximately 90 percent pharmacy participation and a 13 percent return rate within six months (32). Corroborating this, a recent UK study estimated that recycling 2,000 insulin pens could result in carbon savings of 80 kg CO₂e (33). These real-world examples underscore the potential impact of system-level recycling efforts.

## Emerging approaches to include environmental sustainability across the diabetes care pathway and incorporation into HTA decision-making processes

HTA agencies are well-positioned to incentivize sustainable innovation by incorporating environmental considerations into value assessments, guidance, and various decision-making processes along the life cycle of technologies. Nonetheless, at present, there are limited availability of product-level environmental data, a lack of standardized methodologies for quantifying environmental impacts, uncertainty around how to weight environmental outcomes relative to clinical or economic outcomes, and the necessity for multidisciplinary expertise within decision-making processes (7;34–36). Recently, a global survey provided an overview of HTA organizations’ progression toward the integration of environmental sustainability into HTA. The survey investigated various paths for this integration, highlighting potential methods and challenges highlighted by the fact that 9 out of 26 (35 percent) HTA organizations were developing or working toward integrating environmental sustainability into HTA (34).

Incorporating environmental sustainability may require additional analytical expertise and resources, which may not yet be available across HTA organizations. The National Institute for Health and Care Excellence (NICE) highlighted that “*the necessary data and methodological standards for estimating and comparing product-level environmental impacts are not yet mature*” (37). Within the HTA organizational infrastructure, staff may need to be trained or recruited with environmental science and ecological economic expertise, to handle environmental data collection and analysis (7). Nonetheless, it is important to recognize that it is yet to be determined which organization(s) will be responsible for environmental data collection and analysis.

Several approaches have outlined how HTA agencies can incorporate environmental sustainability into decision making, including integrated evaluation, parallel evaluation, the use of information conduits, and environment-focused evaluation (7). An integrated evaluation involves synthesizing and incorporating environmental sustainability and health economic data into a single quantitative value framework. In contrast, a parallel evaluation factors environmental sustainability data into value judgments alongside (evaluated separately) clinical and cost outcomes (7). Ultimately, an integrated and unified approach and methodology to embed environmental sustainability in HTA (ESHTA) (38) would likely provide the most sustainable and valuable approach for healthcare decision making (7).

Integrating environmental assessments into HTA provides valuable insights but faces current challenges, including limited data, methodological complexity, higher resource demands, potential trade-offs with clinical and economic outcomes, and potential delays or equity concerns (7). Therefore, to address the escalating impacts of climate change and health care’s contribution, conducting a parallel assessment with separate thresholds and criteria for environmental versus direct health economic outcomes is useful in the interim, supporting the eventual transition to fully integrated evaluations within HTA.

Several HTA agencies including Scottish Health Technologies Group (SHTG), NICE, the Health Intervention and Technology Assessment Program (HITAP) in Thailand, Institut National d’Excellence en Santé et en Services Sociaux (INESSS) in Quebec, and Canada’s Drug Agency (CDA-AMC) are positioning themselves to undertake a parallel approach (39;40). In 2024, SHTG published the first parallel assessment in Scotland, for throat biopsies (41). This seminal case study demonstrated that evidence of the clinical, cost-effectiveness, and environmental sustainability of the biopsy interventions could be combined into one report. The results showed that the reusable equivalent device was more cost-effective, had patient acceptability, and reduced environmental burden compared to the single-use alternative (41).

In Europe, the European Union HTA Regulation, established to harmonize HTAs across member states, primarily focuses on clinical evaluations while also supporting nonclinical assessments across five domains: economic, ethical, organizational, social, and legal aspects (42). Although environmental sustainability is not explicitly listed, the inclusion of ethical and social domains paves the way for environmental considerations to be factored in (42). Aligned with efforts to integrate environmental sustainability, a recent HTA conducted in Italy by the ALTEMS Advisory research group estimated that introducing a new diabetes technology enabling weekly instead of daily insulin injections could reduce CO₂ emissions by 865 tons over five years (43).

There are precedents for incorporating environmental considerations into HTA evaluation models in other disease areas, although they are mostly limited to considering carbon emissions at present. For example, within asthma management, switching from pressurized metered dose inhaler to dry powder inhaler devices resulted in an estimated 25-fold reduction in carbon emissions (44). As multiple inhaler treatment options are now available for asthma and other respiratory conditions, these innovations, along with product environmental data availability, can empower patients and healthcare professionals to make more informed healthcare decisions. Similar opportunities are emerging in diabetes care, where patient and clinician perspectives increasingly support the integration of environmental sustainability into routine practice. For example, a survey of insulin-treated patients found that although approximately 89 percent expressed at least slight concern about environmental sustainability, 62 percent had never discussed the topic with a healthcare practitioner (45). On the clinician side, a survey of endocrinologists found that 95 percent acknowledged climate change is occurring and 73 percent agreed that climate–health topics should be integrated into medical training (46). The findings indicate both high patient interest and the potential to incorporate environmental sustainability considerations into shared decision making; patient empowerment is a key principle of sustainable health care. However, potential trade-offs and barriers to implementation must be considered. For example, the shift toward increased reuse of medical devices must be supported by infection control policies to ensure clinical safety, while also promoting innovation and the expansion of regional and national decontamination infrastructure (47). In addition, regulatory approval and commissioning processes are considered slow and costly, which can stifle the introduction of innovative sustainable products or ongoing product design improvements (47). Nonetheless, the Green Surgery report highlights real-world initiatives that optimize the current use of resources, embody the principles of sustainable health care, and do not require full use of HTA programs (10). For example, the Intercollegiate Green Theatre Checklist, Compendium of Evidence v2.0, demonstrates how guidance can support healthcare professionals in adopting more sustainable clinical practice and promoting patient empowerment without compromising on patient outcomes (48).

Recognizing some of the challenges and facilitators to support the development of sustainability across the diabetes care pathway, we propose considering the following measures:

- “Health promotion” and wider system change toward diabetes prevention to reduce the overall need for health care (2;10;28). For example, clinical leaders can optimize care pathways, advocate for preventive funding, and, in collaboration with public health, primary care, and local authorities, influence policy and commissioning to support societal change (28).
- Provisioning for and encouraging greater uptake of more sustainable alternatives by patients and health systems (7;23;28) – for example by switching to reusable insulin pens instead of disposable ones, which has been estimated to lead to an 89 percent reduction in plastic waste, a 40 percent reduction in product carbon footprint, and a saving of £75 million pounds in NHS England annually (30).
- Development of new treatments that are more effective at preventing diabetes-related complications, provided there is a holistic consideration of the new treatments’ environmental footprint (29).
- Scaling up innovations which support resource optimization to reduce carbon, plastic, waste, and water footprints downstream (17).
- Development and uptake of treatment options with fewer dispensation requirements such as once-weekly rather than once-daily insulin (43).
- Digitalization of more sustainable administrative processes and technology-enabled care models including digital health solutions, telehealth appointments, and monitoring (42), to reduce the need for patient and staff travel.
- Recycling schemes for disposable insulin pens, integrated into healthcare system infrastructure, previously piloted by Novo Nordisk and Roche (17), Sanofi, Eli Lilly, and Merck (49).

## Future direction and recommendations

HTA organizations have the opportunity to play a pivotal role in advancing sustainable health care, through their capacity to orient action and innovation, toward health promotion, diabetes disease prevention, and more environmentally sustainable practices and interventions (3;4;23). However, progressing sustainability measures within HTA cannot be effectively tackled by HTA agencies in isolation and requires the support of a wider array of policy levers and instruments, such as the development of clinical guidelines that embed environmental considerations alongside safety and efficacy (17;23). Meaningful collaboration across HTA agencies and a wide range of stakeholders, including governments, health systems, HTDs, and the public, is also critical (35). Moreover, health services are working toward net-zero and waste reduction targets, providing further impetus for all stakeholders to improve health technology sustainability. In light of healthcare sustainability targets, prioritizing “greening diabetes” across the entire care pathway (Figure 1) and shifting toward health promotion and disease prevention (10) represent strategic next steps to drive wider system change.

Larger strides across a collaborative HTA landscape include developing policy and guidance to support appropriate reporting of environmental outcomes in HTA. In addition to individual HTA agencies taking steps in this field, international HTA organizations including the International Network of Agencies for HTA (INAHTA) (50) and the HTA International (HTAi) ESHTA Working Group are communicating their positions on incorporating and evaluating ESHTA (36).

Providing a transparent HTA and regulatory landscape for sustainable health innovations is critical to promote more integrated and effective stakeholder collaboration and to incentivize manufacturers and HTD developers by recognizing their environmental sustainability credentials. This may encourage more sustainable innovation and facilitate access to environmentally sustainable interventions across the care pathway. At present, and considering known challenges, a parallel environmental evaluation alongside HTA is suggested to be the most pragmatic and feasible approach to evaluating environmental sustainability. Notwithstanding, we acknowledge that an integrated evaluation approach, where feasible, will optimize the contribution of HTA to sustainable health care by embedding environmental sustainability within the decision-making framework (7).

Methodological tools from other sectors can be used to help evaluate the environmental sustainability of health technologies, including appraising the carbon footprint of a product’s life cycle from raw material acquisition to end of life (Figure 1) (34;51). Notably, the global HTA community is evaluating the feasibility and acceptability of a range of methodologies including environmental life cycle assessment (eLCA), which measures a broader range of environmental outcomes, including climate change, water depletion, water pollution, particulate matter formation, and human ecotoxicity (7;34;51). In response to growing interest in the use of eLCA, several recent studies have illustrated both its applications and the types of insights it can generate in guiding sustainable innovation of technologies (7). However, challenges remain in the application of eLCA in HTA, particularly in relation to data collection; accessing granular data across the full life cycle of a product can be difficult, especially where supply chains are complex or proprietary HTD data are unavailable. In addition, without product category rules (standardized guidelines that specify how eLCAs should be conducted and reported for a particular product category), eLCA results are highly sensitive to methodological choices and risk manipulation, underscoring the need for standardized rules to enable valid product comparisons (52). By publicly disclosing eLCA data, HTDs can empower HTA agencies to integrate environmental outcomes into their evaluations. This integration, while acknowledging jurisdictional variations in HTA priorities and climate conditions, strengthens healthcare sustainability policy and reveals efficiency opportunities for both HTDs and health systems (51).

## Conclusion

The increasing prevalence of diabetes poses a growing challenge to environmental sustainability. However, there are numerous opportunities to integrate sustainable practices across the diabetes care pathway that align with the principles of sustainable health care. Preventing the onset and progression of diabetes through targeted health promotion and disease prevention intervention would allow for a significant reduction in the demand on health services while simultaneously improving the sustainability of diabetes care (16;29). Diabetes therefore stands out as a good area to pilot innovation through targeted approaches, techniques, and tools aimed at integrating environmental sustainability into HTA decision making.

Overall, the inclusion of the environmental sustainability domain in HTA enhances decision making and resource allocation, while simultaneously aligning with sustainability goals, enhancing patient care, and improving population health.
